# Cerebrospinal fluid xanthochromia after pegasparaginase hepatotoxicity in B‐cell acute lymphoblastic leukemia

**DOI:** 10.1002/ccr3.3114

**Published:** 2020-07-16

**Authors:** Riccardo Moia, Mariangela Greco, Paola Boggione, Maura Nicolosi, Riccardo Bruna, Andrea Patriarca, Gianluca Gaidano, Monia Lunghi

**Affiliations:** ^1^ Division of Hematology Department of Translational Medicine Università del Piemonte Orientale Novara Italy

**Keywords:** ALL, CSF xanthochromia, hepatotoxicity, pegasparaginase

## Abstract

Patients with B‐lineage acute lymphoblastic leukemia treated with pegasparaginase‐containing regimens can develop hepatotoxicity related to it. The systemic hyperbilirubinemia due to hepatotoxicity can lead to the development of CSF xanthochromia.

## Graphical abstract

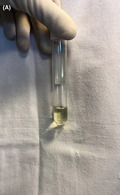

A 44‐year‐old man was referred for a newly diagnosed B‐lineage acute lymphoblastic leukemia.^1^ The patient received a pediatric‐inspired therapeutic regimen, including pegasparaginase. Pegasparaginase administration was complicated by grade 4 hepatotoxicity appearing after 10 days, with bilirubin levels reaching 13 mg/dL and slightly decreasing after 15‐20 days of hydration and l‐carnitine administration.^2^ The timing of appearance and resolution of grade 4 hepatotoxicity is in line with the timing of pegasparaginase‐related hepatotoxicity.^2^ In order to administer intrathecal prophylactic chemotherapy for the prevention of central nervous system recurrence, a preplanned lumbar puncture was performed 32 days after pegasparaginase administration with serum bilirubin levels of 4.9 mg/dL. The lumbar puncture revealed xanthochromia of the cerebrospinal fluid (CSF), as shown in Figure 1, Panel A. Bilirubin in the CSF was positive, with a concentration of 0.1 mg/dL. Lumbar puncture was repeated before cycle 2 (46 days after pegasparaginase infusion with serum bilirubin levels of 2.4 mg/dL) showing a clear liquor (Figure 1, Panel B) and undetectable CSF bilirubin. CSF xanthochromia in this patient reflected the high levels of bilirubin that lasted for more than 15 days in the CSF and may have also accumulated in the central nervous system.

**Figure 1:**
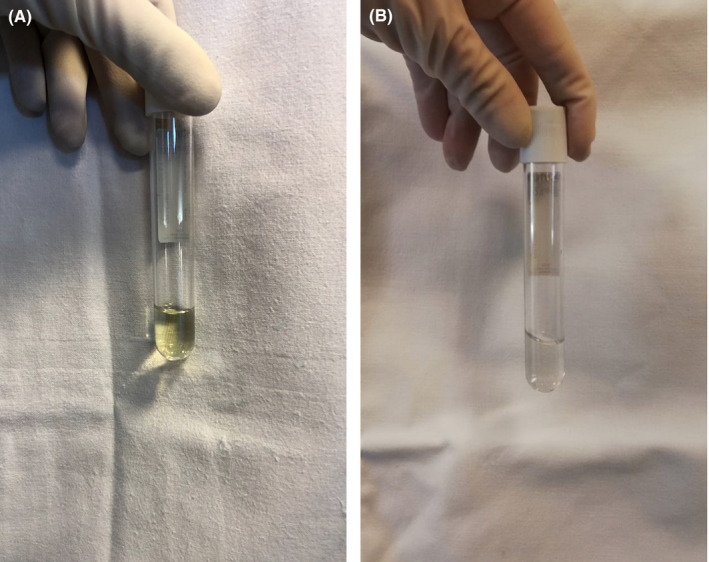
Panel A represents xanthochromia of the CSF. Panel B represents clear CSF after the resolution of pegasparaginase hepatotoxicity

## CONFLICT OF INTEREST

Gianluca Gaidano has to disclose roles in advisory boards or speakers’ bureaus of the following companies: Astra‐Zeneca, Sunesys, Abbvie and Janssen. All the other authors have nothing to disclose.

## AUTHOR CONTRIBUTION

All the authors included in this work contributed to patient care and with manuscript preparation.
